# Equation Córdoba: A Simplified Method for Estimation of Body Fat (ECORE-BF)

**DOI:** 10.3390/ijerph16224529

**Published:** 2019-11-15

**Authors:** Rafael Molina-Luque, Manuel Romero-Saldaña, Carlos Álvarez-Fernández, Miquel Bennasar-Veny, Álvaro Álvarez-López, Guillermo Molina-Recio

**Affiliations:** 1 Department of Nursing, Faculty of Medicine and Nursing, University of Córdoba, Avd Menéndez Pidal No/No, 14004 Córdoba, Spain; rafael.moluq@gmail.com (R.M.-L.); gmrsurf75@gmail.com (G.M.-R.); 2 Department of Occupational Safety and Health, Córdoba City Hall, Huerto de San Pedro el Real, 1, 14003 Córdoba, Spain; craf.19arauco@hotmail.com; 3 Nursing and Physiotherapy Department, Research Group on Evidence, Lifestyles & Health, Research Institute on Health Sciences (IUNICS), Universitat Illes Balears, Carretera de Valldemossa, 5, 07122 Palma, Illes Balears, Spain; miquel.bennasar@uib.es; 4 Hospital Infanta Cristina, Extremadura Health Service, Av. de Elvas, No/No, 06080 Badajoz, Spain; alvarovarez93@gmail.com

**Keywords:** adults, anthropometry, body fat, obesity

## Abstract

*Background:* Many methods for measuring body fat have been developed, but applications in clinical settings are limited. For this reason, researchers have tried to identify different formulas for its estimation but most of are hard to incorporate into daily work due to the variability in population and difficulty of use. The aim of this study was to develop and validate a new equation for the simplified estimation of body fat using the Clínica Universidad de Navarra – Body Adiposity Estimator (CUN-BAE) as a reference. *Methods:* This research was conducted in two phases. In the first, the new body fat estimation equation was developed. The developed equation was validated in the second phase. Pearson’s linear correlation, raw and adjusted linear regressions, the intraclass correlation coefficient, and Bland–Altman graphs were used. *Results:* The variables that best adjusted the body fat percentage were age, sex, and the Napierian logarithm of Body Mass Index (LnBMI), forming the Equation Córdoba for Estimation of Body Fat (ECORE-BF) model. In its validation, the model presented correlation values of 0.994, an intraclass correlation coefficient of 0.960, with the Bland–Altman graph indicating means differences of 1.82 with respect to the estimation with the CUN-BAE. Nevertheless, although the aim was to simplify the CUN-BAE, the main limitation of this study is that a gold standard, such as air displacement plethysmography (ADP) or dual-energy X-ray absorptiometry (DXA), was not used. *Conclusions:* The proposed equation (ECORE-BF) simplified the CUN-BAE and provided a precise method, respecting the principle of parsimony, for the calculation of body fat.

## 1. Introduction

Obesity is defined as being an excess of adipose tissue that may cause health problems [1] and is considered a key risk factor in the development of several chronic diseases such as ischemic heart disease, arterial hypertension, the onset and recovery difficulties of osteo-articular problems, some types of cancer, alterations in the fecundity capacity of men and women, and all-cause mortality [2,3,4,5,6]. The prevalence of being overweight and obesity is continuously increasing, having increased up to 39% and 13%, respectively in 2016 worldwide [7].

Scientific evidence has demonstrated how the amount of body fat and its distribution are influenced by diverse factors, like sex, age, and suffering from certain pathologies or under particular circumstances [8,9,10]. Body fat, both visceral and subcutaneous, is closely related to increased insulin resistance and the subsequent development of metabolic syndrome and type 2 diabetes mellitus [11], which makes it a useful clinical parameter for the prediction and prevention of these diseases.

Several methods are available for measuring body fat, including computerized axial tomography (CT), magnetic resonance imaging (MRI), dual-energy X-ray absorptiometry (DXA), air displacement plethysmography (ADP), and the evaluation of body composition with a four-component model. The latter is based on several techniques (ADP or hydrostatic weighing (UWW), deuterium dilution, and DXA), and is considered to be the gold standard [12,13]. However, given the issues with using these techniques (large-size devices, high costs, use of radiation, etc.), using them for a body composition study in clinical practice, despite their accuracy, is challenging [14,15].

For that reason, researchers have formulated diverse equations for the estimation of different parameters. With regard to body fat percentage (BF%), simple access indexes are available, such as the body mass index (BMI) [16] or the body adiposity index (BAI) [14], among others [17]. Other formulas frequently employed, as they are non-invasive and easy to apply [18], are based on the measurement of body folds. However, deciding which formula to use for its precision and efficiency continues to be a controversial subject both in clinical and research contexts. BMI and BAI are still under debate due to their low correlation with body fat percentage for two reasons: Both do not consider important variables such as age, sex, and because they do not indicate body composition [14,16]. Similarly, due to the large variability in the population to which they are applied, and depending on the professional obtaining the measurements, estimations based on the study of skin folds are also disputed [18,19,20].

Gómez-Ambrosi et al. developed a body fat estimator, the Clínica Universidad de Navarra-Body Adiposity Estimator (CUN-BAE), using ADP, and obtained a correlation of 0.89 with only three variables (age, sex, and BMI) [21]. This formula also achieved good results for the detection of cardiometabolic risk factors when compared with other indexes used for this purpose. CUN-BAE has shown a strong association with metabolic syndrome risk in women (odds ratio (OR) = 6.12) and men (OR = 5.83) [22], arterial hypertension, and type 2 diabetes mellitus [23], which pathologies with a high prevalence [24,25]. As has been demonstrated in other works [26], this estimator is complex due to requiring a calculation with nine components ($(CUN-BAE\;\left(BF\%\right)\;=\;-44.988\;+\;\left(0.503\times Age\right)\;+\;\left(10.689\times Sex\right)\;+\;\left(3.172\times BMI\right)\;-\;\left(0.026\times BMI^{2}\right)\;+\;\left(0.181\times BMI\times Sex\right)\;-\;\left(0.02\times BMI\times Age\right)\;-\;\left(0.005\times BMI^{2}\times Sex\right)\;+\;\left(0.00021\times BMI^{2}\times Age\right)$, in which a man = 0 and a woman = 1 for the sex variable). However, despite the relevance of CUN-BAE, it could be improved for two reasons. Firstly, as highlighted in other research, it is difficult to calculate because it requires nine components. The principle of parsimony, or Ockham’s razor principle, recommends using the simplest model that best fits the measurements. Thus, over-fitting, which means adding more variables to the model, to explain more variability, is considered a methodological limitation [27,28]. However, its complexity means that in clinical real settings, in which software for calculation may not be available, CUN-BAE cannot be used. Since the CUN-BAE is associated with the risk of suffering from the most frequent chronic diseases, its access must be guaranteed in all circumstances, demonstrating the need for the simplest modeling possible, as has been conducted in other populations [29].

For these reasons, the objective of this study was to develop and validate a new body fat estimation equation, reducing the factors required, simplifying the final formula, respecting the principle of parsimony for multivariate modeling, and using the estimations of the CUN-BAE as a reference.

## 2. Materials and Methods

### 2.1. Study Design, Population, and Sample

This research was conducted in two phases. All data were collected from the staff at the City Council of Córdoba (Spain).

#### 2.1.1. Phase I

Phase I was a cross-sectional study with the aim of creating a tool for the estimation of BF% with respect to the reference method (CUN-BAE). The sample (n_1_) was composed of 906 workers selected randomly among those who had underwent an occupational health examination at the City Council Occupational Health Uni, during the period between 2017 and 2019.

#### 2.1.2. Phase II

The purpose of the second phase was to verify the precision of the proposed estimation method. It was performed with a sample (n_2_) of 2000 workers, randomly selected among those employed during the period between 2018 and 2019. The workers included in the one sample did not participate in the other (the n_1_ and n_2_ samples were mutually exclusive).

### 2.2. Study Variables and Measurement

The BF% (result variable) was calculated using the formula proposed by the Clínica Universidad de Navarra, the CUN-BAE [21], and used as the reference method (gold standard):

$$\begin{array}{l} CUN-BAE\;\left(BF\%\right)=\\ \;\;\;\;\;\;\;\;-44.988\;+\;\left(0.503\;\times \;Age\right)\;+\;\left(10.689\;\times \;Sex\right)\;\\ \;\;\;\;\;\;\;\;+\;\left(3.172\times \;BMI\right)\;-\;\left(0.026\;\times \;BMI^{2}\right)\;+\;\left(0.181\;\times \;BMI\;\times \;Sex\right)\;\\ \;\;\;\;\;\;\;\;-\;\left(0.02\;\times \;BMI\;\times \;Age\right)\;-\;\left(0.005\;\times \;BMI^{2}\times Sex\right)\;\\ \;\;\;\;\;\;\;\;+\;\left(0.00021\;\times \;BMI^{2}\times \;Age\right) \end{array}$$

The independent variables collected were: Sex (men and women), age (in years), weight (kg), height (cm), and BMI (kg/m^2^).

The anthropometric measurements were recorded following the recommendations in the standardized anthropometry reference manual [30] by experienced staff to decrease the variation coefficient. Each measurement was recorded three times and the mean value was calculated. The height and weight were measured with a precision of 0.1 cm and 0.1 kg, respectively, using a stadiometer and Atlántida S11 balance (Básculas y Balanzas Añó-Sayol, Barcelona, Spain)

To classify the nutritional state of the study population according to their BMI, the recommendation established by the World Health Organization (WHO) [31] was followed. The sample was categorized in terms of the body fat estimated by the CUN-BAE formula, in accordance with the cut-off points for a Caucasian population [32]: For women: Normal weight, ≤30%; overweight, 30.1%–35%; obese, >35.1%; and Men: normal weight, ≤20%; overweight, 20.1%–25%; obese, >25.1%.

### 2.3. Ethical and Legal Aspects

All the workers were informed, verbally and in writing, of the objectives of the health study to which they were being submitted, and an informed consent was obtained from each in compliance with the current regulations. The study’s protocol complied with the Declaration of Helsinki for conducting medical research involving human subjects and was approved by Bioethics Committee of Córdoba (Spain) (4427/Acta number 295).

### 2.4. Statistical Analysis

The quantitative variables are presented as the mean and standard deviation, the qualitative values presented as frequencies and percentages.

To contrast the goodness-of-fit to a normal distribution of the data from the quantitative variables, the Kolmogorov–Smirnov test with the Lilliefors correction was employed. The Student’s *t*-test for two means was performed for the bivariate hypothesis contrast, whereas for the qualitative variables, the Chi-square and Fisher exact test were used when necessary. In addition, for the correlation between the quantitative variables, the Pearson’s correlation coefficient (*r*) was used.

We used raw linear regressions with each predictive variable and adjusted linear models to obtain new body fat estimation formulas. To determine the goodness-of-fit of the models, we analyzed the standard error, the adjusted coefficient of determination, the F statistic, the linearity analysis, and the residues.

We quantitatively analyzed the degree of concordance with the reference method with the intraclass correlation coefficient (ICC) and, graphically, with the Bland–Altman Method, used the sample n_2_ for this purpose.

For all the statistical analyses, the probability of an α error of below 5% (*p* < 0.05) was considered statistically significant and the confidence interval was calculated at 95%. For the statistical analysis, IBM SPSS Statistics 22.0 software (IBM, Chicago, IL, USA) and Epidat 4.2. (Department of Sanidade, Xunta de Galicia, Galicia, Spain) were used. 

## 3. Results

### 3.1. Prevalence of Overweight and Obesity (n_1_)

Out of a total of 906 workers, 63.1% were men. The mean age was of 42 ± 9.5 years (95% CI: 41.4–42.7 years). The prevalence of obesity following BMI criteria (≥30 kg/m^2^) was 17.5% (95% CI: 15.1%–20.2%), reaching 19.9% (95% CI: 16.7%–23.4%) in men, and 13.5% (95% CI: 10%–17.6%; *p* < 0.001) in women. However, according to the body fat percentage of the CUN-BAE, global obesity prevalence was 53% (95% CI: 49.7%–56.3%), at 57.5% (95% CI: 53.3%–61.6%) in men and 45.2% (95% CI: 39.8%–50.7%) in women (*p* < 0.001). Table 1 summarizes the main characteristics of sample n_1_. 

### 3.2. Bivariate Analysis and Unadjusted Linear Regression

With respect to the BMI categories, statistically significant differences were found between the BMI categories and age, weight, and BF% according to the CUN-BAE (*p* < 0.001). The means of age, weight, and BF% increased from one category to the next, and the means were lower in the underweight category and higher in the obesity category (*p* < 0.001).

A direct correlation between the BF% using CUN-BAE and weight (*r* = 0.273), age (*r* = 0.373), BMI (*r* = 0.640), and its Napierian logarithm (LnBMI) (*r* = 0.625) was observed. We found an inverse correlation with height (*r* = −0.478). All the correlations found were statistically significant (*p* < 0.001).

The correlation between predictive variables and body fat (CUN-BAE) was higher when each sex was analyzed independently, and higher in women than in men, except for BMI (Table 2).

Finally, after performing unadjusted regression models, BMI was the best variable adjusting to CUN-BAE, with a coefficient of determination (*R*^2^) of 0.408, followed by LnBMI (*R*^2^ = 0.390). The determination coefficients were higher for women, except for BMI (Table 3).

### 3.3. Multiple Linear Regression Models (Adjusted) and Clinical Agreement of the Proposed Models

Table 4 presents the multiple linear regression models (adjusted, BF%_n_) obtained for the prediction of body fat percentage together with the correlation and goodness-of-fit of the model with respect to the reference model (CUN-BAE).

The BF%_1_ model explained 98.5% of the variability of the fat percentage estimated by the CUN-BAE formula and has a coefficient of clinical agreement of 0.992 (95% CI: 0.991–0.993). Although the BF%_2_ model explained 95.8% of the variability, the clinical agreement coefficient was 0.625 (95% CI: 0.584–0.663). Similarly, the BF%_3_ model accounted for 97.3% of the variability but its ICC was 0.631 (95% CI: 0.590–0.670). 

When age is incorporated into two models (BF%_2_ and BF%_3_), the equations obtained agreed more with the CUN-BAE, although the variations in the adjusted coefficient of determination are not significant. The BF%_4_ model explained 98.6% of the variability and had a higher correlation and similar agreement to that demonstrated by the first model (0.993; 95% CI: 0.992–0.994). Finally, BF%_5_ best fit CUN-BAE. This model explained a greater variability (99.6%), had better correlation (0.998) and a higher clinical agreement (0.998; 95% CI: 0.997–0.998).

We found the correlation and clinical agreement of sex were reduced in BF%_1_ and BF%_2_ for both men and women. A similar phenomenon occurred with BF%_3_, except that it maintained the correlation at 0.986 for women. The BF%_5_ model remained robust for both measurements, its correlation was not altered and its ICC only diminished by one thousandth (Table 5), which confirms its predictive superiority compared to the other models.

**Figure 1:**
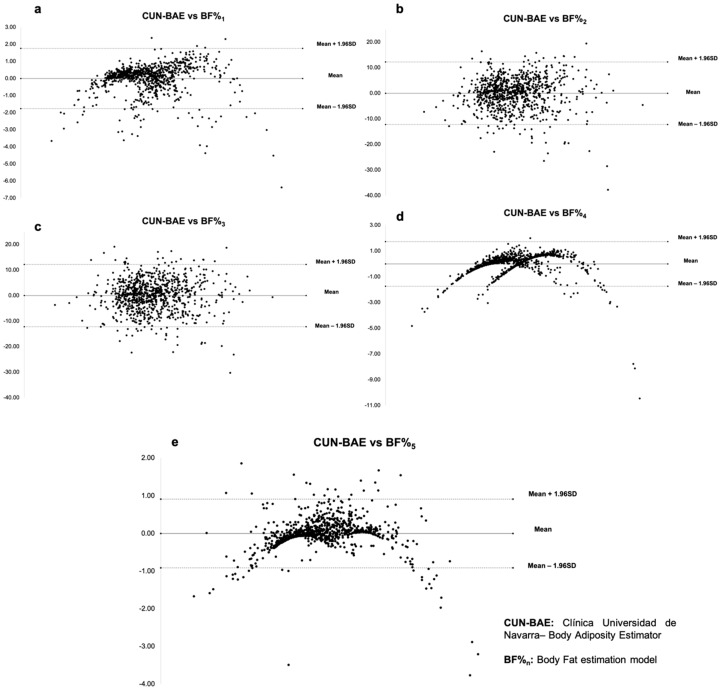
Bland–Altman graphs for body fat estimation models. (**a**) CUN-BAE vs BF%_1_, (**b**) CUN-BAE vs BF%_2_, (**c**) CUN-BAE vs BF%_3_, (**d**) CUN-BAE vs BF%_4_, and (**e**) CUN-BAE vs BF%_5_.

Table 6 and Figure 1 show that according to the Bland–Altman graphs, the model with the greatest clinical concordance is BF%_5_, demonstrating a difference in means and a lower dispersion than the other models.

Depending on sex (Table 6), model BF%_5_ agreed the best with CUN-BAE. As such, the BF%_5_ was considered the best equation for estimating body fat and we called it the Equation Cordoba for Estimation of Body Fat (ECORE-BF).

### 3.4. Validation of the ECORE-FW Method with n_2_ Sample

Of the 2000 workers composing sample n_2_, 1022 (51.1%) were women, with a global mean age of 43.6 ± 10.9 years (95% CI: 43.1–44.0). The mean BMI was 26.7 ± 5.5 g/m^2^ (95% CI: 26.6–27.0). Given the characteristics of n_2_, statistically significant differences were found between sex, age, and BMI with respect to sample n_1_. With respect to the body fat percentage, the mean was 33.7 ± 10.6% (95% CI: 33.2–34.2) and 31.9 ± 8.8% (95% CI: 31.5–32.3) for CUN-BAE and ECORE-BF, respectively.

The correlation obtained between both methods for estimating the body fat percentage (CUN-BAE and ECORE-BF) was 0.994. With regard to sex, the correlation increased to 0.998 for men and women. For the clinical agreement, we observed that the global ICC value was 0.960 (95% CI: 0.957–0.964). For men and women, the ICC was 0.997 (95% CI: 0.997–0.998) and 0.910 (95% CI: 0.897–0.917), respectively. 

**Figure 2:**
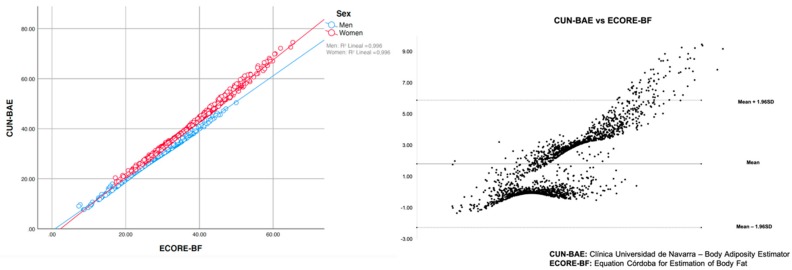
Validation of the proposed model (ECORE-BF: Equation Córdoba for Estimation of Body Fat).

We found a difference in the Bland–Altman agreement means of 1.819 (±2.079), the limits being −2.256 and 5.895. In terms of sex, the difference in means for men was 0.032 (±0.487), with the limits of −0.922 and 0.985, and 3.531 (±1.498) with the limits for 0.595 and 6.470 for women (Figure 2).

## 4. Discussion

The objective of this study was to develop and validate an equation that would simplify the CUN-BAE equation considering the principle of parsimony for multivariate modeling [21].

First, the prevalence of overweight (41.7%) and obesity (17.5%) found in sample n_1_ according to BMI criteria was higher than that presented by the WHO in its latest report [7]. Sex-wise, the men showed a significantly higher proportion of obesity than the women, which has already been observed in a working population [26]. However, the BMI taken as a single reference, as has been reported previously [33], underestimates obesity prevalence as it does not differentiate fat mass from muscle mass [14]. The results show how the obesity prevalence rises to 53% when studied in terms of the percentage of body fat. Regardless, most field studies use BMI as a classification method [34] and it is also employed as an estimator of the body fat percentage [35] due to the good correlation (*r* = 0.640) found in different populations [36].

However, although the BMI and other indexes are used to estimate body fat [17], in their calculation, they do not include important variables like age (*r* = 0.373) and sex [15]. Sex has an increased correlation with all variables, especially BMI *(r* = 0.640 at 0.970 for men and 0.969 for women).

New estimation equations adjusted for sex and age improve the versions that do not include those variables in their adjustment [21]. In this respect, we observed how BMI (BF%_2_ and BF%_4_) improved capacity to explain the variability when age and sex were incorporated (*R*^2^ = 0.408 up to *R*^2^ = 0.986) and had high levels of clinical agreement. Age is the variable least modifying the goodness-of-fit the model evaluated through the adjusted coefficient of determination [26].

Liu et al. [37] published three equations to estimate the body fat in the Chinese population and successfully explained 81.1% of the variability. As with CUN-BAE, it is a complex formula with over six terms and including one more variable, the waist perimeter. Similarly, Kanellakis et al. [38] proposed two methods for the estimation of body fat but, despite possessing good intraclass correlation values (0.955 and 0.976), the formulas presented are complicated and require several anthropometric measurements.

The validation of several estimation formulas in different populations has produced highly variable results in the adjustment with respect to the reference method, obtaining *R*^2^ values between 0.66 and 0.77 [39]. The validation in menopausal women showed similar results, with a high dispersion in the estimation of body fat, and the formulas, including the BMI, explained the variability best over and above skin folds [40]. However, the use of the transformation of the BMI by calculating its Napierian logarithm (LnBMI) significantly improves the fit of the models presented (BF%_3_ and ECORE-BF). Individually, the LnBMI poorly fitted the estimated value of the BF% (*R*^2^ = 0.390) compared with BMI. However, when introducing sex and age into the equation, the explanation of the variability rose to 99.6% and the clinical agreement increased both globally and when differentiated by sex. These values were maintained when applying the formula to another working population with similar characteristics, showing better fits that other studies that used the bioelectrical impedance analysis (BIA) as the gold standard [26]; the latter has not shown a good correlation when using the DXA as a reference test [41].

In all the formulas proposed, the BMI was the variable most influencing the determination of body fat, followed by sex. This changed with the incorporation of LnBMI in which the sex was the variable with more influence (0.788). Thus, this variable increases in importance in body fat determination due to women having 10% more body fat on average [32]. The results established based on standardized β coefficients cannot be compared with other works because they have not been published.

The use of estimation equations may cause various problems as the correlation of the formulas developed vary depending on the reference method used [42] and on the selection of the reference population [34]. For those reasons, as posited by other authors [9], the use of one model or another has to be conditioned by the population studied and by its situation or condition. In addition to the other factors mentioned, the ethnic group may also be a determining factor [13], which complicates the comparison of the results. Furthermore, most of the estimation formulas used in the literature were validated in highly varied populations with distinct characteristics, which complicates their use in current populations [38]. The proposed equation was developed and validated in a population with similar characteristics to that studied by the CUN-BAE, so it is suitable for use in a similar population.

Another issue arising in this study is that a tendency exists to overestimate body fat in slim people and underestimate it in obese people, regardless of the method used [18]. This has been amply corroborated [43] and causes the correlations found to be lower than the most up-to-date values [38]. This situation is aggravated when the overweight or obesity present extreme values, which may affect the CUN-BAE formula as it was calculated by employing the ADP [44] as a reference.

The above evidence demonstrates the need to use equations that best adapt themselves to a specific population with given characteristics (sportspeople, pathologies, slim people, etc.) [9,45,46,47,48,49].

Despite these difficulties, using estimation formulas continues to be a feasible strategy as it guarantees a reduction in the costs and time required to take measurements [45]. In addition, the formulas have shown a good correlation for the detection of the risk of prevalent pathologies and cardiovascular danger factors [16,21,50]. Formulas based on skin folds guarantee a good correlation with respect to DXA [20], but anthropometric measurements, especially in extreme cases like obesity, cause underestimation problems due to their measurement difficulties [51]. However, no formula or method is free from limitations [13,17,52,53,54].

Given the above, we think that employing ECORE-BF is an efficient method for the estimation of body fat could guarantee accuracy, speed, and simplicity. Since only weight and height are required for its calculation in clinical settings, only basic anthropometry skills from healthcare professionals are necessary. However, as a surrogate method was used as a reference for its development and the population with which the formula was validated (individuals with work capacity and aged between 18 and 65 years), these limitations should be highlighted. Despite these limitations, the CUN-BAE showed a high correlation concerning ADP (*r* = 0.89) and a strong association with chronic health problems [21,22,55]. For this reason, the simpler ECORE-BP could be useful when more precise tools are not available.

### Limitations

The main limitation of this study is that a gold standard, such as ADP or DXA, was not used. The use of the CUN-BAE as a reference method does not allow evaluating the real capacity of the proposed method to estimate body fat, due to the limitations of the original equation. Therefore, future work should compare the results obtained by ECORE-BP with more precise methods such as those mentioned. Another limitation of the study is that we found variability in the composition of samples n_1_ and n_2_ in terms of sex, age, and BMI. Nevertheless, the model was shown to be accurate in both samples, even when we found significant differences between them. ECORE-BP not being more accurate on a particular population over another could be one strength of this formula.

## 5. Conclusions

ECORE-BF is a simplification of the CUN-BAE formula that produced precise results with a reduction of the components involved in its calculation. Since ECORE-BF followed the principle of parsimony from multiple regressions, the new formula could be considered as a methodological improvement on CUN-BAE. This simplification is an adaptation of the formula that increased its usability in clinical settings, only requiring basic anthropometry skills from healthcare professionals. ECORE-BF could be applied in settings where more precise tools or specifically trained personnel are not available, or when exposure to radiation must be avoided.

## Figures and Tables

**Table 1:** Description of sample n_1_.

Variable		Total *n* = 906	Men *n* = 572	Women *n* = 334	*p*
		Mean (SD) or *n* (%)	Mean (SD) or *n* (%)	Mean (SD) or *n* (%)	
Age (years)		42 (9.5)	42.3 (9.9)	41.3 (8.7)	0.071
Weight (kg)		75.4 (15)	81.6 (12.9)	64.6 (12.2)	<0.001
Height (m)		168.9 (8.8)	173.4 (6.4)	161.2 (6.5)	<0.001
BMI (kg/m^2^)		26.3 (4.4)	27.1 (3.9)	24.9 (4.7)	<0.001
	Underweight	11 (1.2%)	5 (0.9%)	6 (1.8%)	0.221
	Normal weight	358 (39.5%)	168 (29.4%)	190 (56.9%)	<0.001
	Overweight	378 (41.7%)	285 (49.8%)	93 (27.8%)	<0.001
	Obesity	159 (17.5%)	114 (19.9%)	45 (13.5%)	<0.05
CUN-BAE		29.6 (7.3)	26.4 (5.7)	34.9 (6.5)	<0.001
	Normal weight	137 (15.1%)	58 (10.1%)	79 (23.7%)	<0.001
	Overweight	289 (31.9%)	185 (32.3%)	104 (31.1%)	0.707
	Obesity	480 (53%)	329 (57.5%)	151 (45.2%)	<0.001

Note: BMI, body mass index; CUN-BAE, Clínica Universidad de Navarra-Body Adiposity Estimator.

**Table 2:** Bivariate correlation by sex using CUN-BAE.

Sex	Age	Weight	Height	BMI	LnBMI
Men	0.471 **	0.822 **	−0.115 *	0.970 **	0.977 **
Women	0.541 **	0.868 **	−0.225 **	0.969 **	0.986 **

Note: * *p* < 0.01; ** *p* < 0.001; BMI, body mass index; Ln: Napierian logarithm.

**Table 3:** Raw simple regression.

Global							
Variable	*R* ^2^	Constant	95% CI	Coefficient	SE	95% CI	*p*
Age	0.138	17.589	15.594–19.584	0.285	0.024	0.239–0.332	<0.001
Sex	0.318	26.433	25.948–26.937	8.552	0.415	7.708–9.336	<0.001
Weight	0.074	19.587	17.246–21.928	0.113	0.016	0.102–0.163	<0.001
Height	0.227	96.788	88.708–104.868	−0.398	0.024	−0.446–−0.350	<0.001
BMI	0.408	1.370	−0.874–3.614	1.072	0.043	0.988–1.156	<0.001
LnBMI	0.390	−63.445	−71.044–−55.847	28.562	1.187	26.232–30.892	<0.001
Men							
Age	0.221	14.974	13.160–16.789	0.270	0.021	0.228–0.312	<0.001
Weight	0.675	−3.419	−5.141–−1.696	0.366	0.011	0.345–0.387	<0.001
Height	0.011	44.118	31.542–56.694	−0.102	0.037	−0.174–−0.029	<0.001
BMI	0.940	−12.453	−13.268–−11.637	−1.434	0.015	1.287–1.359	<0.001
LnBMI	0.955	−105.321	−107.677–−102.964	40.046	0.364	39.330–40.761	<0.001
Women							
Age	0.291	18.248	15.387–21.115	0.405	0.035	0.337–0.472	<0.001
Weight	0.752	5.070	3.188–6.951	0.462	0.015	0.433–0.491	<0.001
Height	0.048	71.521	54.453–88.590	−0.227	0.054	−0.333–−0.121	<0.001
BMI	0.939	1.977	1.056–2.897	1.323	0.018	1.287–1.359	<0.001
LnBMI	0.971	−82.439	−84.616–−80.263	36.685	0.345	36.006–37.364	<0.001

Note: BMI, body mass index; Ln: Napierian logarithm; *R*^2^*,* coefficient of determination (goodness of fit); SE, standard error; CI, confidence interval.

**Table 4:** Multiple linear regression models.

Model	Standardized Beta Coefficient		R^2^	SE	*r*	*p*
$\mathrm{BF}{\%}_{1}$ 52.316 + 0.142 (age)+ 11.521 (sex)+ 0.456 (weight) – 0.399 (height)	Age	0.185	0.985	0.888	0.992	<0.001
	Sex	0.763				
	Weight	0.941				
	Height	−0.478				
$\mathrm{BF}{\%}_{2}$ −11.034 + 11.535 (sex) + 1.382 (BMI)	Sex	0.764	0.958	1.496	0.979	<0.001
	BMI	0.824				
$\mathrm{BF}{\%}_{3}$ −100.045 + 11.982 (sex) + 38.442 (LnBMI)	Sex	0.793	0.973	1.202	0.986	<0.001
	LnBMI	0.841				
$\mathrm{BF}{\%}_{4}$ −14.181 + 0.134 (age) + 11.483 (sex) + 1.288 (BMI)	Age	0.176	0.986	0.871	0.993	<0.001
	Sex	0.760				
	BMI	0.768				
$\mathrm{BF}{\%}_{5}$ −97.102 + 0.123 (age) + 11.900 (sex) + 35.959 (LnBMI)	Age	0.161	0.996	0.461	0.998	<0.001
	Sex	0.788				
	LnBMI	0.787				

Note: BF%_n_, body fat estimation model; Sex, men = 0, women = 1; BMI, body mass index; Ln: Napierian logarithm; *R*^2^, coefficient of determination (goodness of fit); SE, standard error; *r*, Pearson’s linear correlation.

**Table 5:** Bivariate correlation and ICC by sex.

Gold Standard	Test	BF%_1_	BF%_2_	BF%_3_	BF%_4_	BF%_5_
Men						
CUN-BAE	*r*	0.991 *	0.970 *	0.977 *	0.993 *	0.998 *
	ICC	0.991 (0.989–0.992)	0.481 (0.415–0.541)	0.492 (0.427–0.551)	0.992 (0.991–0.994)	0.997 (0.996–0.997
Women						
CUN-BAE	*r*	0.987 *	0.969 *	0.986 *	0.985 *	0.998 *
	ICC	0.985 (0.982–0.988)	0.606 (0.533–0.669)	0.600 (0.527–0.664)	0.985 (0.981–0.988)	0.997 (0.996–0.998)

Note: * p < 0.001; 95% CI shown in parentheses; BF%_n_, body fat estimation model; CUN-BAE, Clínica Universidad de Navarra-Body Adiposity Estimator; *r*, Pearson’s linear correlation; ICC, intraclass correlation coefficient.

**Table 6:** Model agreement with CUN-BAE.

Model	Mean Difference (±SD)	*p*	95% CI
Global			
BF%_1_	−0.002 (±0.899)	0.959	−1.763 to 1.760
BF%_2_	0 (±6.249)	1.000	−12.247 to 12.247
BF%_3_	0 (±6.227)	1.000	−12.205 to 12.205
BF%_4_	−0.002 (±0.886)	0.948	−1.739 to 1.735
BF%_5_	−0.000 (±0.466)	0.989	−0.913 to 0.913
Men			
BF%_1_	−0.002 (±0.776)	0.961	−1.523 to 1.519
BF%_2_	−1.351 (±6.226)	1.000	−13.553 to 10.851
BF%_3_	1.298 (±6.138)	1.000	−13.329 to 10.733
BF%_4_	−0.001 (±0.688)	0.977	−1.349 to 1.348
BF%_5_	0 (±0.440)	0.991	−0.863 to 0.863
Women			
BF%_1_	−0.001 (±1.079)	0.981	−2.115 to 2.113
BF%_2_	2.314 (±5.581)	1.000	−8.625 to 12.252
BF%_3_	2.224 (±5.738)	1.000	−9.023 to 13.470
BF%_4_	−0.004 (±1.150)	0.953	−2.258 to 2.251
BF%_5_	−0.001 (±0.507)	0.973	−0.994 to 0.993

Note: BF%_n_, body fat estimation model; CI, confidence interval.
